# Development of a program to determine optimal settings for robot-assisted rehabilitation of the post-stroke paretic upper extremity: a simulation study

**DOI:** 10.1038/s41598-023-34556-3

**Published:** 2023-06-06

**Authors:** Takashi Takebayashi, Yuki Uchiyama, Yuho Okita, Kazuhisa Domen

**Affiliations:** 1 Department of Rehabilitation Science, School of Medicine, Osaka Metropolitan University, 3-7-30, Habikino, Osaka, 583-8555 Japan; 2 Department of Rehabilitation Medicine, Hyogo College of Medicine, Nishinomiya, Hyogo Japan; 3 School of Health Science, Swinburne University of Technology, Melbourne, Australia

**Keywords:** Electrical and electronic engineering, Preclinical research

## Abstract

Robot-assisted therapy can effectively treat upper extremity (UE) paralysis in patients who experience a stroke. Presently, UE, as a training item, is selected according to the severity of the paralysis based on a clinician’s experience. The possibility of objectively selecting robot-assisted training items based on the severity of paralysis was simulated using the two-parameter logistic model item response theory (2PLM-IRT). Sample data were generated using the Monte Carlo method with 300 random cases. This simulation analyzed sample data (categorical data with three difficulty values of 0, 1, and 2 [0: too easy, 1: adequate, and 2: too difficult]) with 71 items per case. First, the most appropriate method was selected to ensure the local independence of the sample data necessary to use 2PLM-IRT. The method was to exclude items with low response probability (maximum response probability) within a pair in the Quality of Compensatory Movement Score (QCM) 1-point item difficulty curve, items with low item information content within a pair in the QCM 1-point item difficulty curve, and items with low item discrimination. Second, 300 cases were analyzed to determine the most appropriate model (one-parameter or two-parameter item response therapy) to be used and the most favored method to establish local independence. We also examined whether robotic training items could be selected according to the severity of paralysis based on the ability of a person (θ) in the sample data as calculated by 2PLM-IRT. Excluding items with low response probability (maximum response probability) in a pair in the categorical data 1-point item difficulty curve was effective in ensuring local independence. Additionally, to ensure local independence, the number of items should be reduced to 61 from 71, indicating that the 2PLM-IRT was an appropriate model. The ability of a person (θ) calculated by 2PLM-IRT suggested that seven training items could be estimated from 300 cases according to severity. This simulation made it possible to objectively estimate the training items according to the severity of paralysis in a sample of approximately 300 cases using this model.

## Introduction

Globally, stroke is a leading cause of physical impairment, with approximately 65% of patients experiencing ongoing post-stroke long-term upper extremity (UE) impairment^1^. Although some patients can regain independence in daily activities within a few months of onset, the majority tend to rely on using only their non-paralyzed hand in daily life^2^. However, some researchers have highlighted the importance of facilitating paralyzed hand use in daily activities to address concerns regarding the decreased quality of life caused by difficulty using the UE^3^.

On the other hand, several researchers reported that appropriate rehabilitation approaches could help improve UE function even in the chronic phase (more than six months after stroke onset) in patients who had a stroke^4,5^. The current stroke guidelines recommend several approaches to post-stroke UE paralysis rehabilitation^6^. Robotic therapy is one of the recommended approaches that allow patients with moderate-to-severe UE paralysis to practice their hand use. This approach can produce outcomes similar to conventional rehabilitation programs^7^. Several studies have used the robotic approach as adjuvant therapy to one-to-one rehabilitation sessions with a physical therapist. We previously conducted a randomized controlled trial (RCT) to investigate the effectiveness of the robotic approach as adjuvant therapy in the subacute and post-stroke phases and found a significant difference in the intervention group^8,9^. However, an RCT from another research group reported no significant UE improvement in patients who had experienced a stroke in a subacute setting compared to either a self-training robot or conventional self-training^10^. Therefore, the effectiveness of robotic therapy as a form of self-training has not been thoroughly clarified. The factors associated with the reported differences in robotic treatment effectiveness in previous studies also remain unclear.

Previous studies have found that several factors are involved in the effectiveness of robot-assisted rehabilitation. It has been suggested that the amount of assistance generated by a robot can affect the intervention outcome^11^. More specifically, when stroke survivors receive excessive robotic assistance, they may be less active in generating voluntary movement of their paralyzed hand, resulting in a so-called ”Slack” state^11^. This study concluded that the group that received no robotic assistance showed more significant improvement in their UE than the group that received excessive robotic assistance^11^. A previous study^10^ provided another example where the control group, who performed exercises with higher intensity compared the intervention group that received the robotic assistance, exhibited better positive outcome. This would indicate that the excessive robotic assistance can cause slacking hypothesis^11^.

Previous studies have reported that minimal robotic assistance can promote post-stroke rehabilitation in patients with moderate-to-severe UE paralysis^12^. Furthermore, some studies have suggested that robotic assistance should be adjusted according to the severity of the stroke survivor’s affected extremity. Specifically, stroke survivors with severe UE paralysis are encouraged to receive increased robotic assistance, while it is suggested that those with moderate-to-light UE paralysis receive less robotic assistance^13,14^. These results suggest that delivering a more optimized intervention with various adjusted parameters, including the ideal amount of robotic assistance, could be key to generating a positive outcome. Additionally, previous research investigated two ways of promoting recovery from post-stroke motor paralysis: facilitating the use of voluntary movements of the paralyzed hand; and minimizing compensatory movements, including the trunk and other body movements associated with paralyzed hand movement^15^. In clinical practice, the optimal setting for robotic assistance often depends on the therapist’s experience and ability to assess the degree of compensatory movements using the trunk rather than the paralyzed hands. Thus, the optimal setting can vary for each therapist.

Item response theory (IRT) is one test theory that can measure the difficulty or discrimination of each assessment item based on the results of the subject's response to a set of items to be evaluated. The main feature of this theory is that it attempts to probabilistically obtain parameters such as individual ability values and each item difficulty (the degree of individual ability and each item difficulty are expressed by the “ability of a person [θ]”) from discrete results such as correct or incorrect responses to assessment items. We evaluated the degree of compensatory movements of stroke patients when practicing each item that the robot has, and analyzed the results with IRT to investigate the possibility of estimating the difficulty level of each item and the degree of individual ability. In addition, a previous study reported that the estimation of item parameters using a two-parameter logistic model based on IRT (2PLM-IRT) requires a sample size of at least 300 cases with an instrument length of a minimum of five items^16^.

Therefore, to select the optimal setting for different abilities of patients with different severities of UE paralysis, we have developed the following research protocol: (1) each patient will try all the severity-specific settings created by combining the parameters of the robot; (2) The therapists will assess the voluntary motor output and degree of compensatory movements for approximately 300 patients in all settings of the robot; (3) we will apply a 2PLM-IRT to the results presented in step 2) above to estimate the difficulty of all items and the ability (θ) of all patients with UE paralysis who participated in this research plan; (4) we will estimate the robot’s items according to the severity of each patient’s paralyzed hand, using, the difficulty level of the item and the ability (θ) estimated in step 3).

However, prior to implementing this research protocol for patients in clinical practice, it is necessary to confirm the feasibility of this study protocol on a sample size of 300 patients which is the maximum number of subjects that can be enrolled in a future clinical study based on simulation data. Therefore, the purpose of this preparatory study is to verify whether a method based on 2PLM-IRT can determine the optimal robot-assisted treatment setting for stroke patients according to the degree of UE paralysis using 300 simulation data generated using random numbers, and to examine the feasibility of several possible analysis methods.

## Methods

### Study protocol

**Figure 1:**
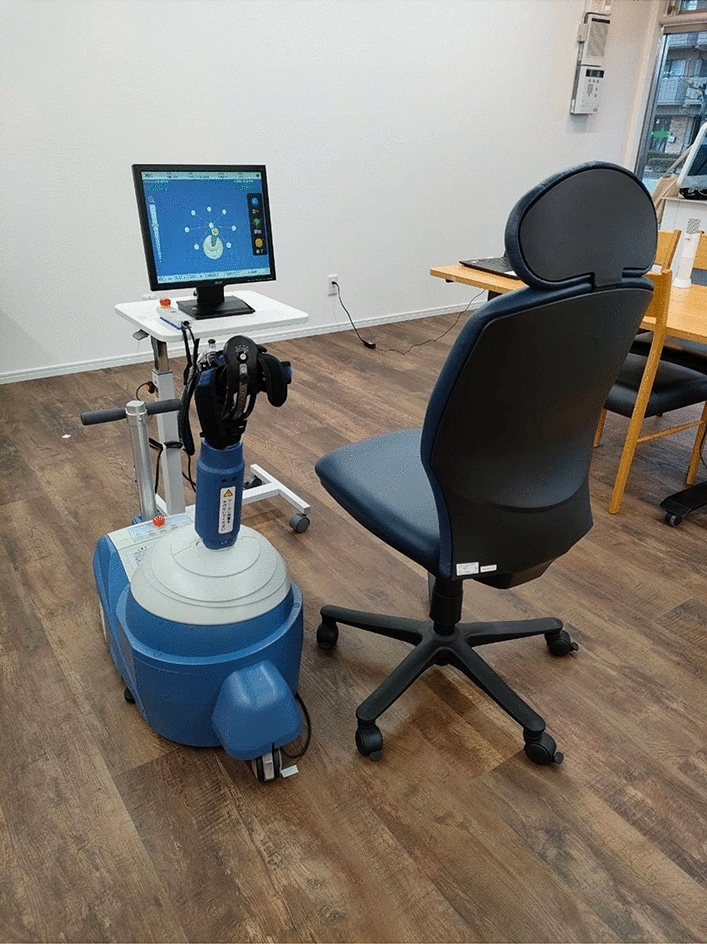
Robot-assisted self-training. The figure illustrates robot-assisted self-training using the ReoGo-J robotic upper extremity rehabilitation device.

**Figure 2:**
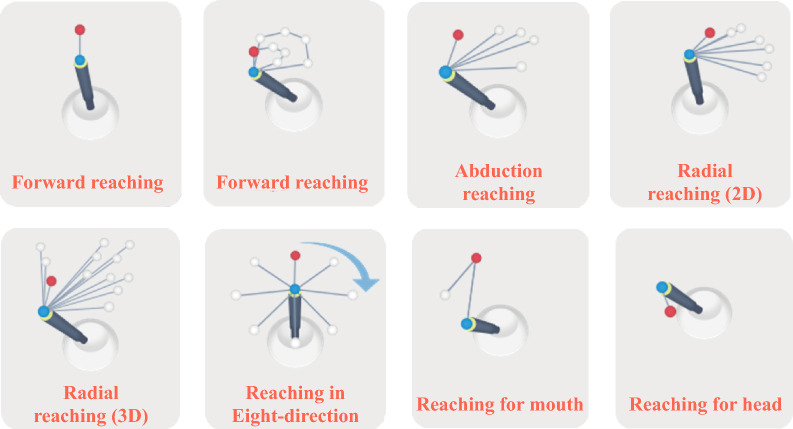
Training items of the ReoGo-J upper extremity rehabilitation device. The ReoGo-J has 17 training tasks. In this study, we select 8 training tasks that used frequently by therapist in past randomized controlled study ^9^.

**Figure 3:**
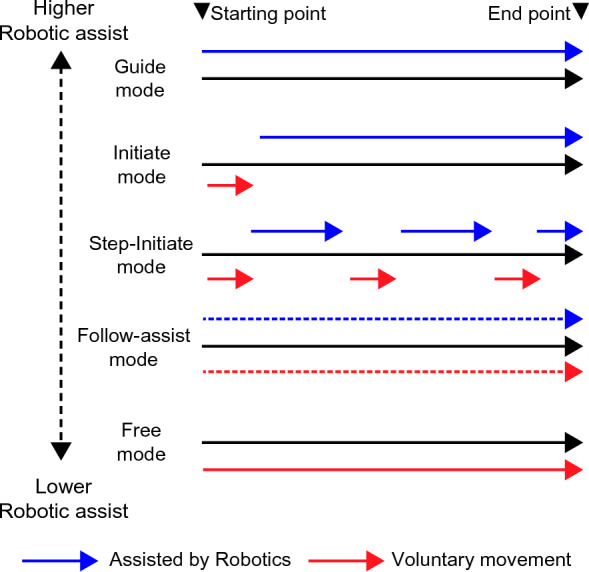
Robotic assistance at the different modes produced by ReoGo-J for voluntary movement. *Guided mode:* fully dependent on robotic assistance to complete training. *Initiated mode:* Requires voluntary movement only at the beginning of training. For the remainder of the training, full dependence on robotic assistance is required. *Step-Initiated mode:* Requires only a few voluntary movements and robot-dependent movements alternately to complete training. *Follow-assist mode:* Required above a certain level of voluntary movement in training while receiving low level of robotic assistance continuously. *Free mode:* Uses voluntary movement to complete training without requiring robotic assistance.

This simulation study aimed to confirm the feasibility of simulated data to determine the optimal robot-assisted treatment setting for stroke patients such that the research findings may be used in the future clinical trial. The same protocol is expected to be used except that the simulated data in this study will be replaced with actual stroke patients’ data in the future clinical trial. In this study, we assumed an intervention using ReoGo-J (Teijin Pharma Limited, Tokyo, Japan). ReoGo-J (Fig. 1) is a robotic system that facilitates functional recovery of the paralyzed hand by allowing stroke survivors to control the robotic arm voluntarily with the paralyzed hand by following instructions displayed on the monitor. The effectiveness of this robot in UE improvement has already been investigated in two RCTs^8,9^. The ReoGo-J system set up training tasks according to the severity of the patient’s paralysis using the following three parameters: (1) Select the appropriate training tasks from eight types of tasks (Fig. 2) that were frequently used by therapist in previous clinical study^9^. The selection was based on an assessment of the degree of freedom of movement in stroke survivors with the severity of their paralysis, and (2) adjust the reach range for each training task considering the severity of the patient’s paralysis (in this research protocol, the default reach range was set at 100, 65, and 30%, with smaller range for the patients with severe paralysis), (3) for patients with moderate-to-severe paralysis, the level of robotic assistance was adjusted to maximize the patient’s voluntary motor output, allowing motorized assisted repetitive movements (Fig. 3) at five levels of assistance. Seventy-one different ReoGo-J settings, combining three parameters, were adopted for the research protocol after consultation with occupational therapy professionals (Supplementary Table 1, Additional File 1).

In the clinical practice of stroke patients, compensatory movements such as using trunk and other parts of the body are frequently used to functionally compensate for disabilities caused by cognitive and physical problems. However, compensatory movements may be an indicator of inappropriate phenomena arising from a number of functional problems that can hinder proper rehabilitation^17,18^. Therefore, it is necessary to monitor compensatory movements to optimize rehabilitation programs for patients with multiple physical problems. Some studies suggest that inhibiting compensatory movements can improve upper limb function^19,20^. In the future clinical study, we will aim to assess the difficulty of the items appropriate to each patient's ability. The specific research method will be used to collect QCM (Quality of Compensatory Movement Score) data to evaluate each compensatory movement by employing patients to perform each of the 71 items of the ReoGo-J. The QCM in this study was evaluated using the following three-case method: QCM 0 points, indicate excessive compensatory movements, including trunk movements and significant difficulty in paralyzed arm training (corresponding to a score of < 3.5 on the Motor Activity Log’s Quality of Movement (QOM) scale^21^ as assessed by the therapist); 1 point, indicates a setting that is neither too difficult nor too easy, the paralyzed hand can complete practice with some effort (corresponding to a score between 3.5 and 4.0 on the Motor Activity Log’s QOM scale as assessed by the therapist); 2 points, indicate a setting where the subject enters a so-called ‘Slack’ state, i.e., the effort to generate voluntary movement is greatly reduced due to excessive robotic assistance (corresponding to > 4.0 points on the Motor Activity Log’s QOM scale as assessed by the therapist). Therefore, we have employed a simulation method using a data set that assumes a three-point method to obtain data for each of the 71 items.

### Method of prepare sample data for simulation studies

The sample data were generated using Monte Carlo methods to generate random numbers using SAS 9.4 for Windows (SAS Institute Inc., Cary, NC, USA) for future clinical trials. The sample data (categorical data with three values [QCM score]: 0, 1, and 2) of 300 cases, with 71 items per case, were analyzed in this simulation. Each of the 71 sample data items was assigned an item number, with higher item numbers indicating a lower QCM score and greater item difficulty. Additionally, we assigned an identification (ID) number to each of the 300 simulated data cases. The purpose of this simulation study was also to verify whether the sample size assumed in this study was sufficient to produce results in future studies. Therefore, the sample size was set at 300, which represents the maximum feasible number of patients for a future clinical study. We created simulated data such that the ability (θ) increased as the ID number value increased. Finally, the cases rated 0 or 2 points in all 71 items were eliminated from the sample data by assuming a scenario where actual patients who experienced stroke used ReoGo-J in the future clinical study.

### Method of analysis

**Figure 4:**
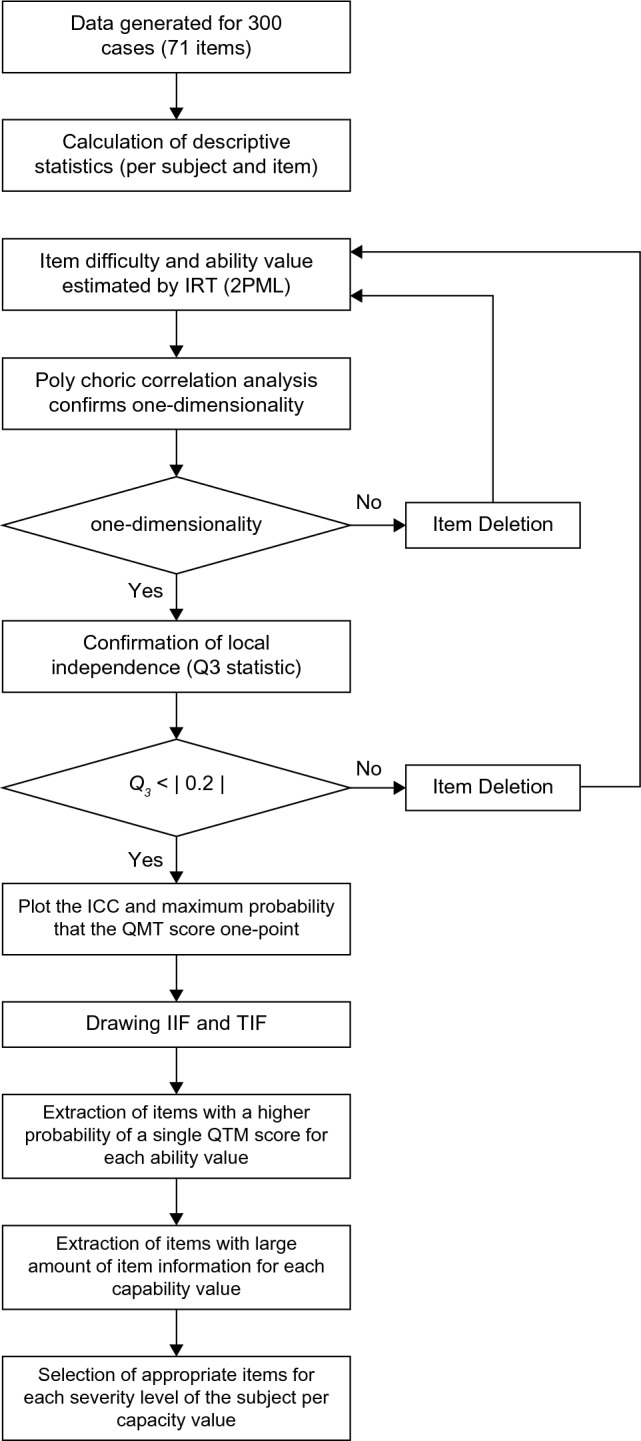
Flowchart: statistical analyses. Flowchart illustrating statistical analyses used in this study.
  For local independence, we calculated the Q3 statistic (i.e., the correlation coefficient of the difference between measured and expected values) as described by Yen et al.^22^ and evaluated whether there were any item pairs for which the absolute value of the Q3 statistic exceeded 0.2. For each item pair exceeding 0.2, one item was excluded from the pair, and the analysis was conducted. The following three strategies including Cronbach’s alpha coefficient, eigenvalues of the polychoric correlation matrix, and values of Akaike information criterion (AIC) and Bayesian information criterion (BIC) (smaller values) were compared to determine the optimal method for item selection, based on the number of pairs exceeding the absolute value of 0.2 in the Q3 statistic. The following rules were applied: (a) exclude items with low response probability (maximum response probability) within a pair in the QCM 1-point item difficulty curve, (b) exclude items with low item information content within a pair in the QCM 1-point item difficulty curve, (c) exclude items with low item discrimination.
  After excluding the relevant items, we also compared the model fit of the statistical findings using the one-parameter logistic model (1PLM) and 2PLM, based on the same criteria as those used for comparing abovementioned strategies. The adequacy of the model’fit was assessed by comparing its AIC and BIC values. The model that exhibited the smallest AIC and BIC values out of all the models and strategies was deemed relatively adequate, and adopted.

1. Analysis before addressing local independence
  Figure 4 shows the statistical procedures used to address the local independence. The item parameters were estimated using 2PLM- IRT, while the marginal maximum likelihood (MML) estimation method was used to estimate each item’s discriminative power and difficulty level. The ability (θ) in each case was estimated using the MML estimation method, assuming a standard normal distribution. The item parameters and ability (θ) were estimated using the 2PLM-IRT procedure in SAS. Parallel analysis was performed on the poly correlation coefficients to confirm the unidimensionality of each item.

2. Analysis after addressing local independence
  Figure 4 shows the statistical procedures performed after addressing the local independence. The cases identified after addressing local independence were removed for the simulated data, and the item parameters were estimated using 2PLM-IRT. The MML estimation method was used to estimate the discriminative power and difficulty level of each item. The ability (θ) in all cases was estimated using the MML estimation method, assuming a standard normal distribution.
  Parallel analysis was performed on the poly correlation coefficients to confirm the unidimensionality of each item. For local independence, we calculated the Q3 statistic (correlation coefficient of the difference between the measured and expected values) for the remaining 61 items, as described by Yen et al.^22^. We evaluated whether there were any item pairs for which the absolute value of Q3 exceeded 0.2. A scatter plot was created with the estimated ability (θ) set on the horizontal axis and the mean value of the QCM score on the vertical axis, to discriminate monotonicity. Each graph was created to represent the following: item characteristic curve (ICC), ICC for a single QCM point per item, item information function (IIF), and test information function (TIF).

3. Identification of the optimal item for each ability of a person (θ) in the patient

1. Identification based on the response probability criterion with a 1-point QCT:
  For each target item to be analyzed, the probability of 1-point QCT was calculated using $p_{j1} (\theta ) = p_{j1} { \star }(\theta ) - p_{j2} { \star }(\theta ),pjc{ \star }(\theta ) = 1/\{ 1 + \exp [ - Daj(\theta - bjc{ \star })]\}$. We identified the item with the largest response probability of a 1-point QCT along with the ability (θ) by substituting each item parameter into this equation. Additionally, for each ability (θ), we examined the top seven items with the highest probability of 1-point response on the QCT.

2) Identification based on the item information criterion:
  The information content of an item is calculated by $I_{j} (\theta ) = D^{2} \alpha_{j}^{2}$ $\sum\limits_{ c = 0}^{c - 1} {\frac{{\left\{ {pjc{ \star }\left( \theta \right)qjc{ \star }\left( \theta \right) - pjc + 1{ \star }\left( \theta \right)qjc + 1{ \star }\left( \theta \right)} \right\}2}}{ pjc\left( \theta \right)}}$. We examined the top seven items for each ability (θ) by substituting the parameters estimated from the MML estimator into this equation and changing the value of (θ).

3) Comparison of identification methods based on response probability criteria with the QCM score of 1 point and each item information criterion:

In terms of each criterion, we defined and investigated the top seven items with high values from the response probability criteria with a QCM score of 1 point and item information criterion as follows: severe, − 2.0 ≤ θ < − 0.5; moderate, − 0.5 ≤ θ < 0.5; and mild, 0.5 ≤ θ < 2.0. The analysis for the entire range of − 2.0 ≤ θ < 2.0 was conducted simultaneously. The analysis was performed by incorporating the relevant items into the 2PLM- IRT for each condition (severity domain and identification method). We assessed the adequacy of the model’s fit within each range by comparing their AIC and BIC values. The models that exhibited smaller AIC and BIC values were considered superior in two criteria. After comparing both criteria across four ranges, we determined that the criterion with the highest number of superior models was the most appropriate choice. All statistical analyses in this study were performed using SAS 9.4 and Microsoft excel 2016 for Windows.

## Results

### Simulation results of 71 items before addressing local independence (Analysis before addressing Local Independence)

The descriptive statistics of the QCM data for the 300 simulated cases had a mean value of 0.99, standard deviation of 0.64, minimum value of 0.03, median value of 1.01, and maximum value of 1.99. The average QCM scores for each item (71 items) had average values of 0.99, 0.30, 0.30, 0.48, 1.01, and 1.49, respectively.

With the increasing identification (ID) value of the case numbers (sample data), the frequency of appearance of the 0 points QCM score decreased, two points increased, and one point emerged almost consistently, regardless of the item number. Furthermore, one point appeared at the highest rate when the ID of the case was approximately 150. In the estimation of item parameters, the mean values of item discrimination and item difficulty 1.2 was 2.928, − 0.436, and 0.449, respectively. The estimated value of item difficulty ([item difficulty 1 + item difficulty 2]/2) was 0.005. Item difficulty increased with the ID value of the item. The ability of a person (θ) was as follows: mean value − 0.001; standard deviation, 0.984; minimum value − 1.897; median, 0.007; and maximum, 2.064. The ability (θ) increased with a higher ID value of the case number in the target cases.

To confirm the model assumption, the unidimensionality was confirmed by poly correlation analysis, which showed that the first eigenvalue in the poly correlation matrix was 56.094, and the second eigenvalue was 0.75. Since the second eigenvalue was less than 1.0, one-dimensionality was considered established. To confirm local independence, 10 item pairs (item ID: 70–71, 38–49, 1–19, 59–69, 5–19, 12–30, 13–28, 50–70, 8–29, 13–46) had an absolute Q3 value exceeding 0.2. When comparing the three strategies presented in the Methods section, strategy (a) that excluded items with low response probability (maximum response probability) within a pair in the QCM 1-point item difficulty curve, appeared to be the most suitable decision method for establishing local independence (The excluded ID numbers were 1, 5, 12, 28, 29, 38, 46, 50, 69, and 70). Furthermore, the model fit between the 1PLM and 2PLM, showing that 2PLM was the ideal model for data analysis after the removal of 10 items (Table 1).

**Table 1:** Comparison between three strategies and two models in item response theory, for adopting the best method for selecting deleted items to establish local independence.

Evaluation method	Two-parameter logistic model				one parameter logistic model		
	Strategies for establishing local independence*						
	None	Strategy 1	Strategy 2	Strategy 3	none	Strategy 1	Strategy 2
Number of items analyzed^†^	71	61	63	63	71	62	64
Number of pairs (Q3 statistic > |0.2|)	10	0	1	1	9	1	2
Cronbach α	0.991	0.990	0.990	0.990	0.991	0.990	0.991
Polychoric correlation matrix (Value of first eigenvalue)	56.1	48.2	49.9	49.3	56.1	49.0	50.7
The polychoric correlation matrix (Rate of first eigenvalue)	0.790	0.791	0.793	0.792	0.790	0.790	0.793
AIC^††^	25,930	22,484	23,071	23,055	25,845	22,756	23,412
BIC^††^	26,719	23,162	23,771	23,771	26,374	23,219	23,890
Comparison model fitting^‡^	×	○	×	×	×	×	×

*Strategy 1; QCM score 1 point: Exclude items with low 1-point response probability (maximum probability) when the QCM score is 1 point in item difficulty; Strategy 2: Exclude items with limited item content information when the QCM score is 1 point in item difficulty; Strategy 3: Exclude items with low item discrimination.

^†^The reason for the difference in the analyzed items for each strategy is that the target pairs included duplicate items.

^††^Smaller AIC and BIC values can be interpreted as higher model fitting.

^‡^The adequacy of the model’s fit was assessed by comparing its AIC and BIC values. The model that exhibited the smallest AIC and BIC values out of all the models and strategies was deemed relatively adequate.

○Relatively good: The model fit that exhibited the smallest BIC and AIC values among the models and strategies analyzed.

× Relatively bad: The model fit that exhibited smaller BIC and AIC values than the model or strategy indicated by the circle icon.

*AIC* Akaike’s information criterion, *BIC* Bayesian information criterion, *QCM* Quality of Compensatory Movement Score.

### Simulation results of 61 items after addressing local independence (Analysis before addressing Local Independence)

Ten items were deleted from the 71 items to preserve local independence using strategy (a), resulting in 61 items being analyzed. The descriptive statistics of the QCM data for the 300 simulated cases were as follows: mean value 0.99, standard deviation 0.65, minimum value 0.03, median value 1.00, and maximum 1.98. The QCM score for each item (61 items) had a mean value of 0.99, standard deviation of 0.30, minimum value of 0.48, median value of 1.01, and a maximum value of 1.45. Overall, these results were almost identical to the results including all 71 items.

As in the analysis of all 71 items, the frequency of appearance of the 0 points QCM score decreased with increasing case number ID, 2 points increased, and 1 point emerged almost consistently regardless of item number. Furthermore, one point appeared to be the most frequent closest to ID values of approximately 150.

**Figure 5:**
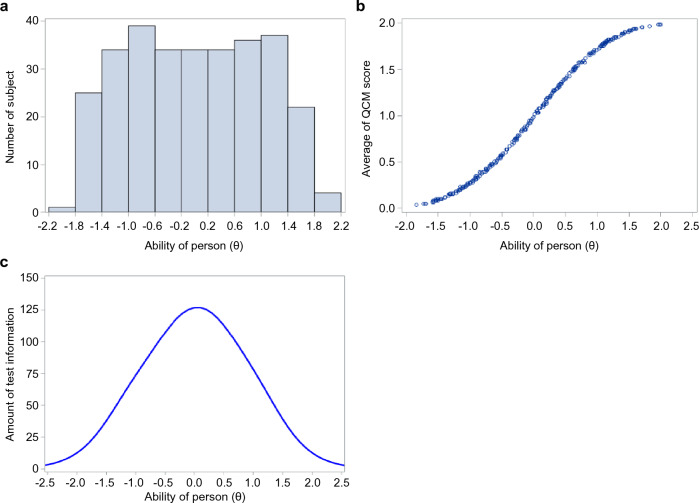
Distribution chart of various data. (**a**) Variance of sample data in this study simulated by Monte Carlo method. (**b**) Confirmation of monotonicity between quality of movement scores and the ability of a person for each sample data. (**c**) Distribution of item information across 61 items.

In the estimation of the item parameters, the mean values of item discrimination and item difficulty 1.2 were 2.931, − 0.436, and 0.450, respectively. The estimated value of item difficulty ([item difficulty 1 + item difficulty 2]/2) was 0.007. Item difficulty increased with the ID value of the item. The ability of a person (θ) was as follows: mean value, 0.0000; standard deviation, 0.981; minimum value − 1.839; median, 0.007; and maximum, 1.998. The ability (θ) increased with a higher ID number in the target cases. Although the distribution of the ability (θ) was originally expected to be normally distributed, the actual distribution is left-skewed, as illustrated in Fig. 5a.

To confirm the model assumption, unidimensionality was confirmed by poly correlation analysis: the first eigenvalue in the poly correlation matrix was 48.244, and the second was 0.64; since the second eigenvalue was less than 1.0, one-dimensionality was considered established, ensuring unidimensionality. Local independence was confirmed as there were no pairs for which the absolute value of the Q3 statistic exceeded 2. Monotonicity was confirmed as the mean value of the QCM increased almost monotonically, corresponding to an increase in the ability (θ) (Fig. 5b).

The apex of the curve of item probability in QCM score 1 was found to shift toward the direction of the higher ability (θ) with higher values of item ID. This finding suggests that the difficulty of the item increased with a higher ID value.

The apex of each item’s curve was found to shift toward the direction of the higher ability (θ) with higher item ID values. This result suggests that the ability (θ) to provide the maximum amount of information (optimal measurement accuracy) for an item increases with a higher value of ID. The item information functions for all 61 items are shown in Fig. 5c. This figure shows that the measurement accuracy and test information content of the sample data are maximized when the ability (θ) is approximately zero.

### Selection of the optimal item ID for the ability of a person (θ) of each subject

1. Discrimination based on the reaction probability criterion for the 1 point QCM score

**Figure 6:**
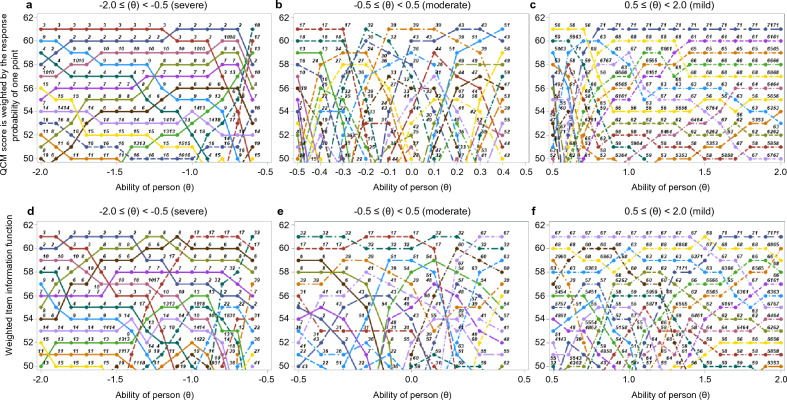
Selection of the appropriate items for each ability of a person (θ) in sample data. In the severe (**a**), moderate (**b**), or mild group (**c**), seven items were selected that had a relatively high probability of scoring 1 on the QCM score. In the severe (**d**), moderate (**e**), or mild group (**f**), seven items were selected that had a relatively high item information function.

The top seven items with the highest probability of obtaining a QCM score of one point for each ability (θ) in the cases are shown in Fig. 6a–c. In the range of the ability (θ) from − 2.0 ≦ θ < − 0.5 (severe), the response probability of each item varied, but the items with the highest response probability were item ID2, 3, 6, 7, 8, 9, and 10 (Fig. 6a). In the range − 0.5 ≦ θ < 0.5 (moderate), the response probability of each item varied with no discernible pattern, but the items with the highest response probability were items ID 30, 32, 33, 36, 39, 43, and 44 (Fig. 6b). In the range from 0.5 ≦ θ < 2.0 (mild), the response probability of each item varied, but the items with the highest response probability were items ID56, 61, 63, 65, 66, 68, and 71 (Fig. 6c).

2. Discrimination by the item information criterion

The top seven items with high item information content for each ability (θ) are shown in Fig. 6d–f. In the range of the ability (θ) from − 2.0 ≦ θ < − 0.5 (severe), the response probability of each item varied significantly, but the items with the highest response probability were items ID 2, 6, 7, 8, 10, 13, and 17 (Fig. 6d). In the range from − 0.5 ≦ θ < 0.5 (moderate), the response probability of each item was relatively consistent, but the items with the highest response probability were items ID 17, 31, 32, 39, 41, 43, and 51(Fig. 6e). In the range of 0.5 ≦ θ < 2.0 (mild), the response probability of each item varied significantly, but the items with the highest response probability were items ID60, 62, 63, 66, 67, 68, and 71 (Fig. 6f).

3. Comparison of discrimination methods based on response probability criteria with the QCM score of 1 point and the item information criterion

The results of the 2PLM- IRT for each condition are shown in Table 2. The following results were found: the discrimination method based on the QCM score of 1 point was relativity superior in − 2.0 ≤ θ < − 0.5 range; the discrimination method based on the item information criterion was relatively superior in both − 0.5 ≤ θ < 0.5, 0.5 ≤ θ < 2.0, and − 0.2 < θ < 2.0 ranges. The model fit of the item information criterion was relatively superior to the QCM score of 1 points criterion in three of the four ranges.

**Table 2:** Comparison of models based on item response theory.

Classification of the ability of a person (θ)* (Severity)	− 2.0 ≦ θ < − 0.5 (Severe)		− 0.5 ≦ θ < − 0.5 (Moderate)		0.5 ≦ θ < 2.0 (Mild)		− 2.0 ≦ θ < 2.0 (All patients)	
Criterion for selection**	Strategy 1	Strategy 2	Strategy 1	Strategy 2	Strategy 1	Strategy 2	Strategy 1	Strategy 2
Number of items	7	7	7	7	7	7	7	7
Value of items	2, 3, 6, 7, 8, 9, 10	2, 6, 7, 8, 10, 13, 17	30, 32, 33, 36, 39, 43, 44	17, 31, 32, 39, 41, 43, 51	56, 61, 63, 65, 66, 68, 71	60, 62 63, 66, 67, 68, 71	2, 3, 17, 39, 43, 56, 71	2, 6, 8, 17, 32, 67, 71
Evaluation method								
Cronbach α	0.922	0.929	0.940	0.942	0.915	0.927	0.916	0.916
Polychoric correlation matrix (Value of first eigenvalue)	5.578	5.706	5.842	5.948	5.474	5.686	5.755	5.966
Polychoric correlation matrix (Rate of first eigenvalue)	0.797	0.815	0.835	0.850	0.782	0.812	0.822	0.852
AIC^†^	2920	2930	3189	3096	2939	2815	3028	2847
BIC^†^	2998	3009	3266	3174	3016	2893	3106	2925
Comparison model fitting^††^	○	×	×	○	×	○	×	○

*The ability of a person (θ): a measure of ability to be measured by item response theory. It represents the estimated ability of an individual examinee, and its mean value is 0 for all examinees.

**Strategy 1: discrimination based on the reaction probability criterion for the QCM score of 1 point; strategy 2: discrimination by the item information criterion.

^†^Smaller AIC and BIC values can be interpreted as higher model fitting.

^††^The adequacy of the model’s fit within each range was assessed by comparing its AIC and BIC values. The models that exhibited smaller AIC and BIC values were considered superior. Additionally, after comparing both criteria across four ranges, we determined that the criterion with the highest number of superior models was the most appropriate choice for assessing goodness of fit.

○Relatively good: The model fit that exhibited smaller BIC and AIC values compared between the strategy 1 and 2 in each range.

× Relatively bad: The model fit that exhibited larger BIC and AIC values compared between the strategy 1 and 2.

*AIC* Akaike’s information criterion, *BIC* Bayesian information criterion, *QCM* Quality of Compensatory Movement Score.

## Discussion

In this study, a simulation method was employed to investigate the feasibility of estimating the item difficulty for each training item setting and the corresponding ability of a person (θ). This study applied 2PLM-IRT and evaluated the QCM scores of 71 different training items in ReoGo-J. Our results showed that ReoGo-J training items could be set at the optimal difficulty level for each subject without being affected by assumed characteristics of the 300-sample data, which was simulated with the most likely variance in stroke patients using Monte Carlo methods. This result could be because the application of the 2PLM-IRT allowed separate modeling of the ability of a person (θ) in the patient and item difficulty, as well as discrimination for each of the 71 items. Furthermore, this indicates that patients with a higher ability (θ) were more likely to adapt to items with higher difficulty. In addition, the 2PLM-IRT used in this analysis assumes local independence in its theoretical construction. This simulation was performed on data from 300 cases generated using random numbers. To establish local independence, ten items according to the ability of a person (θ) were removed from the 71 items. As a sample size for 2-PLMIRT, 300 cases has been shown to be adequate in previous studies^16^, and the results appeared to be applicable to our future clinical studies.

Regarding the concept of task difficulty in motor learning, Guadagnoli et al.^23^ stated that it is necessary to select a task with optimal difficulty for the subject’s ability to facilitate motor learning, even if subjects with different skill levels are given the same training tasks. In a study using machine learning, Wilson et al.^24^ reported that the optimal difficulty level for learning a new concept is approximately 85% (neither too easy or difficult) of correct answers (success rate), considering the ability of the subject. Similarly, our study’s findings appeared to follow this learning theory. This suggests that simulation results can be used to help adjust the difficulty of items in practice using a robot according to the ability of a person (θ). More specifically, this simulation study confirmed the possibility of setting the optimal difficulty for seven out of the 61 items with local independence in ReoGo-J, considering the ability (θ) for each patient. This was confirmed based on the response probability criterion with a 1-point QCM score and the discrimination method based on the item information content criterion. Therefore, it is assumed that the optimal training task can be selected in a real clinical setting if the ability (θ) can be evaluated.

Next, this study confirmed the best method to select the optimal seven training tasks that correspond to the ability (θ). This was completed by comparing two methods: a discrimination method of the item response probability criterion for the QCM score of 1 point and the discrimination method based on the item information criterion. Our results suggest that the discrimination method based on the item information criterion is more likely to help select the optimal training task to set for each subject than that of the response probability criterion with a QCM score of one point.

Furthermore, in the discrimination method based on the item information criterion, the fit of the 2PLM-IRT was confirmed in the analysis based on three severity (moderate, − 0.5 ≦ θ < − 0.5; mild, 0.5 ≦ θ < 2.0) and all subjects (− 2.0 ≦ θ < 2.0). Among severe subjects with − 2.0 < θ < − 0.5, the QCM score of 1 points criterion had smaller AIC and BIC value than the item information criterion. However, when considering the smaller AIC and BIC values as true values, the relative error between the two values was 0.33–0.34%, indicating little difference between the two criteria. Therefore, in our view, the 2PLM-IRT was found to be compatible across all levels of severity in the discrimination method, as determined by the item information criterion.

However, since only seven training tasks could be applied to all cases with varying severity of UE paralysis, the selected model may not translate well to a clinical scenario. Based on the results of these simulations, it seems reasonable to use the item information criterion discriminant method, segregated by UE paresis severity using the ability (θ), when conducting analyses using clinical data. However, the results of this simulation study showed that the discriminant method based on item information criteria had a better model fit than that based on item response probability criteria with the score 1 QCM. However, we assume that the model fit concept can change depending on the data variance. Therefore, it is necessary to re-examine the goodness-of-fit of the model when applying it to clinical data. Additionally, if the model adaptability of the two discrimination methods appears low when using clinical data, it would be necessary to check the model adaptability of items that can be seen in both discrimination methods.

If the procedure of this simulation study is to be used in clinical practice, it is necessary to assess the ability of a person (θ) after stroke to assess the severity of paralysis when selecting items, although it is difficult to accurately estimate the ability (θ) for stroke patients. However, Woodbury et al.^25^ suggested a gold standard outcome measure for functional impairment of UE paralysis, and the Fugl-Meyer Assessment (FMA) for UE function can be used to categorize the severity of impairment (severe: 0–19 points; moderate: 20–46 points; mild: 47 points). Additionally, it may be necessary to evaluate the FMA score for UE in each patient and estimate the relationship between the FMA score for UE and the ability (θ) using a correlation formula to accurately estimate the ability (θ) in future clinical studies.

There are several limitations in this study. Since this was a simulation study, it is yet to be investigated whether the characteristics of compensatory movement characteristics in the 71 ReoGo-J specific items affect item selection. However, future clinical studies will explore the compensatory movement characteristics specific to the ReoGo-J and incorporate them into the algorithm for selecting appropriate items based on subjects’ abilities. Second, as is a simulation study, it is unclear how the 71 ReoGo-J-specific items can affect their ADL performance. Future clinical studies are expected to evaluate the relationship between the characteristics of the ReoGo-J tasks and ADLs performance, and to develop an algorithm that not only extracts appropriate items based on the subject's ability, but also estimates the ADLs performance that would be acquired as a result. Third, throughout this simulation, we encountered difficulties adapting the algorithm to account for cognitive decline that might interfer with the item selection in the robotic practice. Our next clinical trial is expected to recruit only those with sufficient cognitive function, ROM, stiffness to participate in robotic practice.

## Supplementary Information

Supplementary Information.

## Data Availability

The datasets are available from the corresponding author on reasonable request.

## Abbreviations

AIC: Akaike information criterion
BIC: Bayesian information criterion
FMA: Fugl-Meyer Assessment
IIF: Item information function
IRT: Item response theory
MML: Marginal maximum likelihood
QCM: Quality of Compensatory Movement
RCT: Randomized controlled trial
TIF: Test information function
UE: Upper extremity
