# Supplementary material for: Development of a program to determine optimal settings for robot-assisted rehabilitation of the post-stroke paretic upper extremity: a simulation study
Source: Sci Rep. 2023 Jun 6;13:9217. doi: 10.1038/s41598-023-34556-3 (PMC10244345; doi:10.1038/s41598-023-34556-3)
Supplement: Supplementary file 1 — Supplementary Information. [file 41598_2023_34556_MOESM1_ESM.doc]

Supplementary Table 1. Training items of ReoGo-J

The actual practice items were programmed into ReoGo-J (the order of each item is not associated with the difficulty level).

| Variation of task | Training mode | Range of reaching (%) |  | Variation of task | Training mode | Range of reaching (%) |
| --- | --- | --- | --- | --- | --- | --- |
| Forward reaching | Initiate | 65 |  | Radial reaching (2D) | Follow-assist | 100 |
| Forward reaching | Step-initiate | 30 |  | Radial reaching (2D) | Free | 30 |
| Forward reaching | Step-initiate | 65 |  | Radial reaching(2D) | Free | 65 |
| Forward reaching | Step-initiate | 100 |  | Radial reaching (2D) | Free | 100 |
| Forward reaching | Follow-assist | 30 |  | Radial reaching (3D) | Initiate | 65 |
| Forward reaching | Follow-assist | 65 |  | Radial reaching (3D) | Step-initiate | 30 |
| Forward reaching | Follow-assist | 100 |  | Radial reaching (3D) | Step-initiate | 65 |
| Forward reaching | Free | 30 |  | Radial reaching (3D) | Step-initiate | 100 |
| Forward reaching | Free | 65 |  | Radial reaching (3D) | Follow-assist | 30 |
| Forward reaching | Free | 100 |  | Radial reaching (3D) | Follow-assist | 65 |
| Rotation reaching | Initiate | 65 |  | Radial reaching (3D) | Follow-assist | 100 |
| Rotation reaching | Step-initiate | 30 |  | Reaching in eight-direction | Initiate | 65 |
| Rotation reaching | Step-initiate | 65 |  | Reaching in eight-direction | Step-initiate | 30 |
| Rotation reaching | Step-initiate | 100 |  | Reaching in eight-direction | Step-initiate | 65 |
| Rotation reaching | Follow-assist | 30 |  | Reaching in eight-direction | Step-initiate | 100 |
| Rotation reaching | Follow-assist | 65 |  | Reaching in eight-direction | Follow-assist | 30 |
| Rotation reaching | Follow-assist | 100 |  | Reaching in eight-direction | Follow-assist | 65 |
| Rotation reaching | Free | 30 |  | Reaching in eight-direction | Follow-assist | 100 |
| Rotation reaching | Free | 65 |  | Reaching in eight-direction | Free | 30 |
| Rotation reaching | Free | 100 |  | Reaching in eight-direction | Free | 65 |
| Abduction reaching | Initiate | 65 |  | Reaching in eight-direction | Free | 100 |
| Abduction reaching | Step-initiate | 30 |  | Reaching for mouth | Initiate | 65 |
| Abduction reaching | Step-initiate | 65 |  | Reaching for mouth | Step-initiate | 30 |
| Abduction reaching | Step-initiate | 100 |  | Reaching for mouth | Step-initiate | 65 |
| Abduction reaching | Follow-assist | 30 |  | Reaching for mouth | Step-initiate | 100 |
| Abduction reaching | Follow-assist | 65 |  | Reaching for mouth | Follow-assist | 30 |
| Abduction reaching | Follow-assist | 100 |  | Reaching for mouth | Follow-assist | 65 |
| Abduction reaching | Free | 30 |  | Reaching for mouth | Follow-assist | 100 |
| Abduction reaching | Free | 65 |  | Reaching for head | Initiate | 65 |
| Abduction reaching | Free | 100 |  | Reaching for head | Step-initiate | 30 |
| Radial reaching (2D) | Initiate | 65 |  | Reaching for head | Step-initiate | 65 |
| Radial reaching (2D) | Step-initiate | 30 |  | Reaching for head | Step-initiate | 100 |
| Radial reaching (2D) | Step-initiate | 65 |  | Reaching for head | Follow-assist | 30 |
| Radial reaching (2D) | Step-initiate | 100 |  | Reaching for head | Follow-assist | 65 |
| Radial reaching (2D) | Follow-assist | 30 |  | Reaching for head | Follow-assist | 100 |
| Radial reaching (2D) | Follow-assist | 65 |  |  |  |  |
